# Derivation of the economic value of R_0_ for macroparasitic diseases and application to sea lice in salmon

**DOI:** 10.1186/s12711-018-0418-6

**Published:** 2018-10-03

**Authors:** Kasper Janssen, Hans Komen, Helmut W. Saatkamp, Mart C. M. de Jong, Piter Bijma

**Affiliations:** 1 Animal Breeding and Genomics, Wageningen University and Research, P.O. Box 338, 6708 PB Wageningen, The Netherlands; 2 Business Economics, Wageningen University and Research, P.O. Box 8130, 6706 KN Wageningen, The Netherlands; 3 Quantitative Veterinary Epidemiology, Wageningen University and Research, P.O. Box 338, 6708 PB Wageningen, The Netherlands

## Abstract

**Background:**

Macroparasites, such as ticks, lice, and helminths, are a concern in livestock and aquaculture production, and can be controlled by genetic improvement of the host population. Genetic improvement should aim at reducing the rate at which parasites spread across the farmed population. This rate is determined by the basic reproduction ratio, i.e. ${\text{R}}_{0}$, which is the appropriate breeding goal trait. This study aims at providing a method to derive the economic value of ${\text{R}}_{0}$.

**Methods:**

Costs of a disease are the sum of production losses and expenditures on disease control. Genetic improvement of ${\text{R}}_{0}$ lowers the loss-expenditure frontier. Its economic effect depends on whether the management strategy is optimized or not. The economic value may be derived either from the reduction in losses with constant expenditures or from the reduction in expenditures with constant losses.

**Results:**

When ${\text{R}}_{0}$ ≤ 1, the economic value of a further reduction is zero because there is no risk of a major epidemic. When ${\text{R}}_{0}$ > 1 and management is optimized, the economic value increases with decreasing values of ${\text{R}}_{0}$, because both the mean number of parasites per host and frequency of treatments decrease at an increasing rate when ${\text{R}}_{0}$ decreases. When ${\text{R}}_{0}$ > 1 and management is not optimized, the economic value depends on whether genetic improvement is used for reducing expenditures or losses. For sea lice in salmon, the economic value depends on a reduction in expenditures with constant losses, and is estimated to be 0.065€/unit ${\text{R}}_{0}$/kg production.

**Discussion:**

Response to selection for measures of disease prevalence cannot be predicted from quantitative genetic theory alone. Moreover, many studies fail to address the issue of whether genetic improvement results in reduced losses or expenditures. Using ${\text{R}}_{0}$ as the breeding goal trait, weighed by its appropriate economic value, avoids these issues.

**Conclusion:**

When management is optimized, the economic value increases with decreasing values of ${\text{R}}_{0}$ (until the threshold of ${\text{R}}_{0} = 1$, where it drops to zero). When management is not optimized, the economic value depends on whether genetic improvement is used for reduced expenditures or production losses. For sea lice in salmon, the economic value is estimated to be 0.065 €/unit ${\text{R}}_{0}$/kg production.

**Electronic supplementary material:**

The online version of this article (10.1186/s12711-018-0418-6) contains supplementary material, which is available to authorized users.

## Background

Macroparasites, such as ticks, lice, and helminths, are a concern in livestock and aquaculture production worldwide. Macroparasites may reduce the wellbeing of the animals [1], are transmitted from livestock to humans [2], impose a threat to wild populations due to pathogen spillover from farmed animals [3, 4], and induce economic costs in farming [5, 6]. Free range (outdoor) farming is particularly prone to macroparasites compared to indoor farming, because generally it provides more favourable conditions for parasites to complete their lifecycle, and thus the risk of infection from wild populations may be higher [2, 7]. The worldwide trend from free-range farming to indoor farming of monogastrics [8] could have reduced parasite prevalence globally, while the opposite trend in farming conditions that occurs in some developed countries [9] might increase parasite prevalence locally. For the same reasons that free-range farming is more prone to macroparasites than indoor farming, outdoor cage and pond aquaculture—the dominant forms of aquaculture—are likely to be more prone to macroparasites than indoor aquaculture.

The prevalence of macroparasites is controlled by (1) preventive measures that minimize the risk of infection, inhibit the rate at which parasites spread, and interrupt the parasitic lifecycle, and (2) by treatment with drugs or other methods. Treatment efficacy tends to deteriorate over time as parasites often evolve drug resistance [10]. Because of the evolution of drug-resistant parasites and the stringent regulations on maximum residue limits for drugs, control of parasites is increasingly difficult [11]. The rate at which parasites evolve drug resistance is expected to increase as the frequency of treatments increases, and when treatment relies only on a few treatment mechanisms compared to a combination of various treatment mechanisms (e.g. drugs and temperature treatment). Based on the same principle, genetic improvement of farm animals may be more sustainable when selection is performed on many underlying loci with small effects compared to selection on a single quantitative trait locus with a large effect [12]. Nevertheless, both simulation studies [12] and empirical evidence [13] suggest that parasite evolution will not revert the effect of genetic improvement of livestock in the short term. Thus, genetic improvement of resistance to macroparasites in farm animals is a highly desirable addition to the repertoire of control measures.

Genetic improvement should aim at reducing the rate at which parasites spread across the farmed population, and combined with the management strategy, this rate determines prevalence. This rate is determined by the basic reproduction ratio, ${\text{R}}_{0}$, which was previously proposed as the appropriate breeding goal trait for infectious diseases by Anche et al. [14]. For macroparasitic diseases, ${\text{R}}_{0}$ is defined as “the average number of offspring (female offspring in a dioecious species) that are produced throughout the reproductive lifespan of a mature parasite and that survive to reproductive maturity in the absence of density-dependent constraints on population growth” [15], where density-dependent constraints refer to parasite density. In the absence of density-dependent constraints, ${\text{R}}_{0}$ is an “exponential” per parasite generation growth factor for the number of parasites per host. ${\text{R}}_{0}$ has a threshold value of 1. When ${\text{R}}_{0}$ > 1 and density-dependent constraints are absent, the parasite population can grow. When ${\text{R}}_{0} < 1$, the parasite population declines after initial infection and no major epidemic can occur. ${\text{R}}_{0}$ is a widely used parameter in epidemiology to describe macroparasitic infections using the definition above, and to describe microparasitic infections in e.g. a susceptible–infected–recovered (SIR) model. For both macro-and microparasitic infections, ${\text{R}}_{0}$ combines susceptibility, infectivity, contact rate, and recovery rate in a single parameter [15, 16]. Today, selection by breeding companies aims mostly at reducing susceptibility, while methods to estimate infectivity are being developed [17, 18]. Other traits of potential interest for genetic improvement include tolerance and resilience, and we discuss their relevance later in the paper.

For optimal implementation in breeding programs, the economic value of ${\text{R}}_{0}$ should be known. The economic value of a trait is a linear approximation of the change in farm profit due to a one unit change in the trait from its current value [19]. Economic values are needed to maximize economic gain. However, no method has been developed for the derivation of the economic value of ${\text{R}}_{0}$ for macroparasites, or alternatively for the derivation of economic values of susceptibility and infectivity. This study presents a method for the derivation of the economic value of ${\text{R}}_{0}$ for macroparasitic diseases. The method is specific to macroparasites that are the causative pathogen. It does not apply to macroparasites that act as a vector of microparasitic diseases, such as Lyme disease, because the transmission dynamics are different and production losses are determined by the microparasite rather than the macroparasite.

First, we describe the effect of improvement in ${\text{R}}_{0}$ on farm profit via reduced production losses and/or reduced expenditures. Then, we consider these effects for situations where management is or is not optimized. We determine the effect of improvement of ${\text{R}}_{0}$ when expenditures or losses are kept constant and provide algebra to derive the economic value. A numerical example is provided for illustration, and we apply the method to find the economic value of ${\text{R}}_{0}$ for sea lice in Atlantic salmon. Finally, we discuss implications and limitations of the method.

## Methods

### Effect of R_0_ on farm profit

The economic value of ${\text{R}}_{0}$ is a linear approximation of the change in farm profit due to a one unit change in ${\text{R}}_{0}$ from its current value:

$${\text{EV}} = \frac{{\partial {\text{Profit}}}}{{\partial {\text{R}}_{0} }}.$$

To derive the economic value, we are interested in the change in profit per unit change in ${\text{R}}_{0}$, rather than in its absolute level. Because a reduction in ${\text{R}}_{0}$ increases farm profit, the economic value is negative. However, for presentation purposes, we shall ignore the minus sign in the economic value throughout the remainder of the text.

**Fig. 1:**
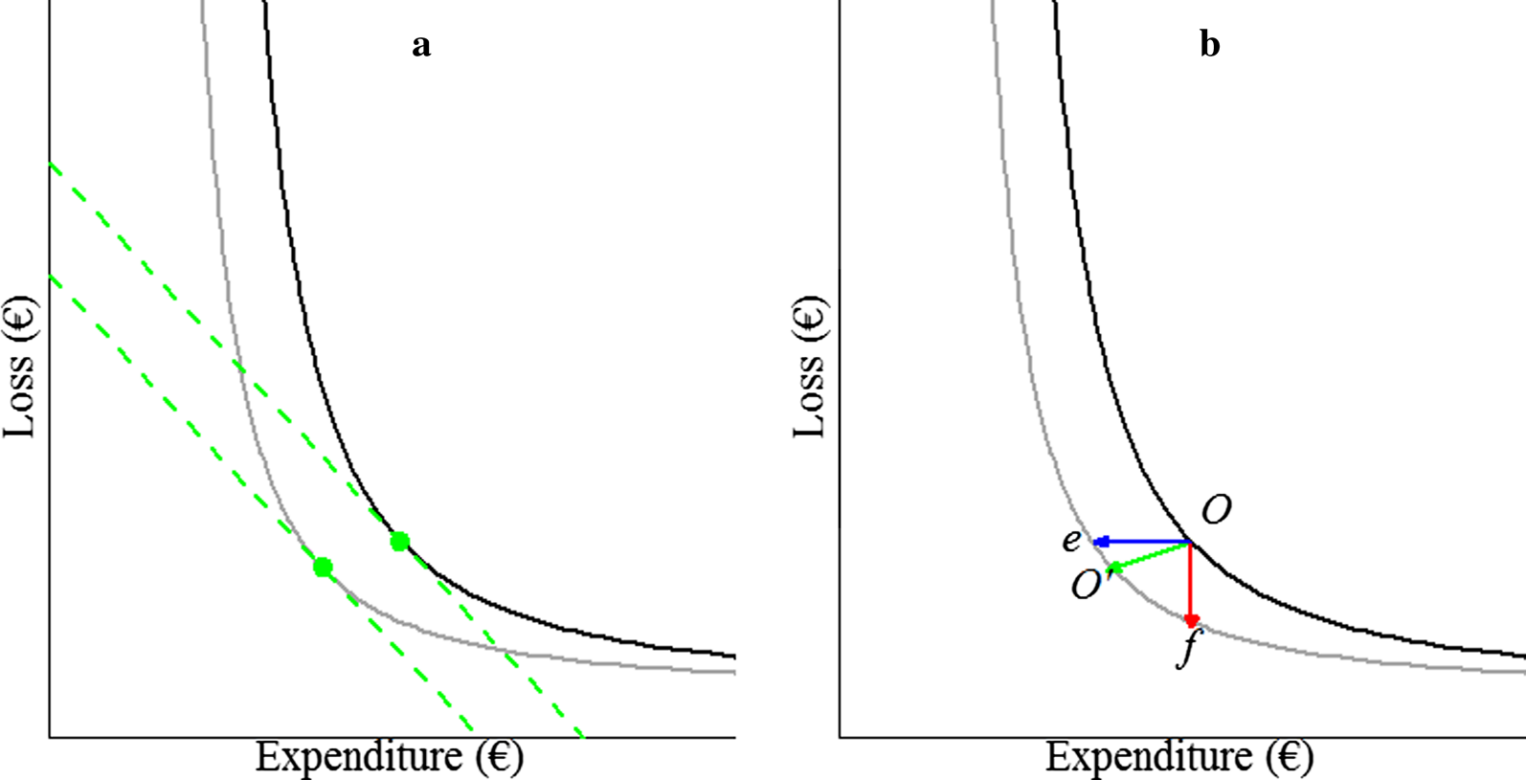
Loss-expenditure frontiers (solid curves) for two values of ${\text{R}}_{0}$, with the frontier on top having the highest ${\text{R}}_{0}$. **a** Green dots: economic optima, dashed lines: ∂Loss⁄∂Expenditure = − 1. **b** Reduction in cost due to a reduction in ${\text{R}}_{0}$. Green arrow: reduction in losses and expenditures when moving from optimum *O* to optimum *O′*, blue arrow ending in *e*: reduction in expenditures at constant losses, red arrow ending in *f*: reduction in losses at constant expenditures

Costs of livestock diseases are the sum of production losses (${\text{L}}$) and expenditures on control (${\text{E}}$) [20]. ${\text{L}}$ are reduced revenues, e.g. due to decreased productivity or reduced product quality, and ${\text{E}}$ are costs of treatment and prevention. The loss-expenditure frontier gives the minimum level of ${\text{L}}$ for any level of ${\text{E}}$, both expressed in monetary units. ${\text{L}}$ decreases when ${\text{E}}$ increases, because expenditures on disease control reduce production losses. The sum of ${\text{L}}$ and ${\text{E}}$ takes a concave shape. Figure 1a shows the hypothetical loss-expenditure frontiers for two values of ${\text{R}}_{0}$. For any given frontier, the value of ${\text{R}}_{0}$ is constant. All farmers operate on or above the loss-expenditure frontier. In economics, farmers that operate on the frontier are referred to as “efficient”, while farmers that operate above the frontier are not. Different levels of ${\text{E}}$ reflect differences in disease control management. Some farmers may choose to have a low ${\text{E}}$ and incur a relatively high ${\text{L}}$ as a consequence, whereas other farmers may choose for a high ${\text{E}}$ and incur a relatively low ${\text{L}}$. Farmers that operate at the level of ${\text{E}}$ at which costs are minimum are at the economic optimum. The economic optimum is reached when the sum of ${\text{L}}$ and ${\text{E}}$ is minimal. At this optimum, the sum of ${\text{L}}$ and ${\text{E}}$ does not change with a marginal change in ${\text{E}}$: $\frac{{\partial \left( {{\text{L}} + {\text{E}}} \right)}}{{\partial {\text{E}}}} = 0$ or equivalently $\frac{{\partial {\text{L}}}}{{\partial {\text{E}}}} = - 1$. Genetic improvement of ${\text{R}}_{0}$ lowers the loss-expenditure frontier and thereby reduces ${\text{L}}$, ${\text{E}}$, or both, for farmers that are efficient both before and after genetic improvement. Here, we focus on the economic value of ${\text{R}}_{0}$ for efficient farmers; benefits for inefficient farmers depend on how they capitalize on genetic gains.

### Optimized management

In livestock genetic improvement, it is common to derive economic values in the context of optimized management, because improvement of management is generally easier to achieve than genetic improvement. The management variable ${\text{E}}$ is a function of ${\text{R}}_{0}$, and ${\text{E}}$ and ${\text{R}}_{0}$ together determine ${\text{L}}$. Thus, ${\text{E}}$ should be at the economic optimum before genetic improvement [21], which results in the optimum level of L. The optimum before genetic improvement is at point *O* in Fig. 1b. Following improvement of ${\text{R}}_{0}$, the initial optimum *O* will move to a new optimum *O*′ between points *e* and *f*. For a small (infinitesimal) improvement in ${\text{R}}_{0}$, the new loss-expenditure frontier between points *e* and *f* can be approximated linearly by $\partial {\text{L}}/\partial {\text{E}} = - 1$. Thus, the sum of $\partial {\text{L}}$ and $\partial {\text{E}}$ when moving from the initial optimum *O* to any point on the lower frontier between *e* and *f* is constant. Moving to any point between *e* and *f* on the lower frontier therefore gives the same cost reduction. When ${\text{E}}$ is optimized before genetic improvement, the economic value (${\text{EV}}$) may be derived either as the partial derivative of ${\text{L}}$ with respect to ${\text{R}}_{0}$ while ${\text{E}}$ is held constant:

1 $${\text{EV}} = \frac{{\partial {\text{L}}}}{{\partial {\text{R}}_{0} }} ,$$

or as the partial derivative of ${\text{E}}$ with respect to ${\text{R}}_{0}$ while ${\text{L}}$ is held constant:

2 $${\text{EV}} = \frac{{\partial {\text{E}}}}{{\partial {\text{R}}_{0} }} .$$

For an infinitesimal change in ${\text{R}}_{0}$, both methods give the same result as when moving from the optimum level of expenditures (point *O*) before genetic improvement to the optimum level of expenditures (point *O′*) after genetic improvement.

### Non-optimized management

According to neoclassical economic theory, with no constraints on either ${\text{L}}$ or ${\text{E}}$, farm management converges to the economic optimum strategy. However, in reality farmers may constrain either ${\text{L}}$ or ${\text{E}}$ for non-economic reasons or for economic reasons that are not apparent from the costs of the disease. For example, farmers may keep ${\text{E}}$ at a constant level below its optimum, because of restrictions on the use of antibiotics. Conversely, farmers may keep ${\text{E}}$ above its optimum, because a minimum frequency of treatments is enforced by legislation. Similarly, farmers may keep ${\text{L}}$ below its optimum for animal welfare reasons. Thus, it may be relevant to derive the economic value for a situation where management is not optimized, and where the deviation from the optimum originates from constraints on either ${\text{L}}$ or ${\text{E}}$. In other words, constraints on either ${\text{L}}$ or ${\text{E}}$ may push farmers to operate at a suboptimum level of ${\text{E}}$. Here, we assume that these constraints will remain after genetic improvement.

When ${\text{E}}$ is constrained either below or above its optimum, genetic improvement of ${\text{R}}_{0}$ will reduce ${\text{L}}$ while ${\text{E}}$ is kept constant. For this scenario, the economic value can thus be derived as the reduction in ${\text{L}}$ per unit change in ${\text{R}}_{0}$ while ${\text{E}}$ is kept constant (Eq. 1), denoted by the red arrow in Fig. 1b.

When ${\text{L}}$ is constrained below its economic optimum, as in the animal welfare example, genetic improvement of ${\text{R}}_{0}$ will reduce ${\text{E}}$ while ${\text{L}}$ is kept constant. For this scenario, the economic value can thus be derived as the reduction in ${\text{E}}$ per unit change in ${\text{R}}_{0}$ while ${\text{L}}$ is kept constant (Eq. 2), denoted by the blue arrow in Fig. 1b. We consider situations where ${\text{L}}$ is constrained above its optimum as irrelevant, because we cannot think of a realistic example. Note that in the antibiotics example, the level of ${\text{L}}$ is above its optimum, but this follows from a constraint on ${\text{E}}$ rather than ${\text{L}}$, and improvement of R_0_ will reduce ${\text{L}}$ with constant ${\text{E}}$.

### Derivation of the economic value when expenditures are kept constant

When expenditures are kept constant, the economic value follows from Eq. 1. Thus, in this case, we need to express ${\text{L}}$ as a function of ${\text{R}}_{0}$. ${\text{L}}$ has been broadly defined as production losses but is defined more specifically as lost production due to parasites, which equals the deviation of the actual production in the presence of parasites relative to the production that would have been achieved in the absence of parasites. In our definition, ${\text{L}}$ is independent of the production level itself. Here, we assume that ${\text{L}}$ is linearly related to the mean number of parasites per host [22]. Hence, production losses per host per parasite are assumed constant. This assumption implies that given the mean, variation in the number of parasites per host within the herd and over time can be ignored when deriving the economic value. Thus, to define ${\text{L}}$ as a function of ${\text{R}}_{0}$, we need to define the mean number of parasites per host as a function of ${\text{R}}_{0}$.

We assume that minimum and maximum numbers of parasites in animal production are controlled within such a narrow range that the growth of the number of parasites per host within this range is exponential and can be described by the growth factor per parasite generation, ${\text{R}}_{0}$. Thus, when ${\text{R}}_{0}$ > 1, the number of parasites per host grows exponentially over time until treatment is applied. We assume that treatment reduces the number of parasites per host to a fixed minimum, i.e. ${\text{I}}_{ \min }$. Because a single treatment involves a fixed expenditure, the interval between treatments remains constant when ${\text{E}}$ is kept constant, such that the number of parasite generations between two treatments also remains constant. This implies that, for a given ${\text{I}}_{ \min }$ and a given number of parasite generations between treatments, the mean number of parasites per host between treatments is determined by ${\text{R}}_{0}$. The transition of one production cycle to the next may affect the number of parasites per host. The relative importance of this effect is diluted when the length of a production cycle is long compared to the period between treatments. Here, we assume that the mean number of parasites per host over the total length of a production cycle can be approximated by the mean number of parasites per host between two treatments. Because ${\text{L}}$ is proportional to the mean number of parasites per host over the length of a production cycle, ${\text{L}}$ can be expressed as a function of ${\text{R}}_{0}$. The corresponding algebra is provided below.

Let ${\text{I}}_{ \min }$ be the minimum number of parasites per host and $\uptau$ the number of parasite generations between two treatments. The number of parasites per host (${\text{I}}$) over parasite generations (${\text{t}}$) is a function of ${\text{R}}_{0}$ as:

3 $${\text{I}}\left( {{\text{R}}_{0} ,{\text{t}}} \right) = {\text{I}}_{ \min } \cdot {\text{R}}_{0}^{\text{t}} .$$

The mean number of parasites per host over a period of $\uptau$ generations between treatments is:

4 $${\bar{\text{I}}}\left( {{\text{R}}_{0} ,\uptau} \right) = \frac{{\mathop \smallint \nolimits_{0}^{\tau } {\text{I}}\left( {{\text{R}}_{0} ,{\text{t}}} \right){\text{dt}}}}{\uptau} = \left( {\frac{{{\text{I}}_{\min } \cdot {\text{R}}_{0}^{\uptau} }}{{\ln \left( {{\text{R}}_{0} } \right)}} - \frac{{{\text{I}}_{\min } \cdot {\text{R}}_{0}^{0} }}{{\ln \left( {{\text{R}}_{0} } \right)}}} \right) \cdot \frac{1}{\uptau} = \frac{{{\text{I}}_{\min } \cdot \left( {{\text{R}}_{0}^{\uptau} - 1} \right)}}{{\uptau \cdot \ln \left( {{\text{R}}_{0} } \right)}} ,$$

which increases with ${\text{R}}_{0}$ and $\uptau$. Note that *τ* ≠ *t*. Let ${\text{T}}$ be the length of a production cycle in parasite generations, and $\bar{\text{I}}\left( {{\text{R}}_{0},\text{T}} \right)$ the mean number of parasites per host over period ${\text{T}}$. We assume that ${\text{T}} \gg\uptau$, which allows us to ignore the effect of the transition from one production cycle to the next. Thus, $\bar{\text{I}}\left( {{\text{R}}_{0},\text{T}} \right)$ is approximated by $\bar{\text{I}}\left( {{\text{R}}_{0},\uptau} \right)$.

Let ${\text{L}}_{\text{par}}$ be the production losses (e.g. in euro) per host per parasite present over a period of ${\text{T}}$ parasite generations. The change in the level of a production trait per parasite is known as tolerance or the slope of a reaction norm, and is the linear regression of the production trait on the number of parasites per host [23]. Thus, ${\text{L}}_{\text{par}}$ is defined as:

5 $${\text{L}}_{\text{par}} = \frac{{\partial {\text{profit}}}}{{\partial {\bar{\text{I}}}\left( {{\text{R}}_{0} } \right)}} = \mathop \sum \limits_{{{\text{i}} = 1}}^{\text{n}} \left( {\frac{{\partial {\text{trait}}_{\text{i}} }}{{\partial {\bar{\text{I}}}\left( {{\text{R}}_{0} ,{\text{T}}} \right)}} \cdot \frac{{\partial {\text{profit}}}}{{\partial {\text{trait}}_{\text{i}} }}} \right) ,$$

where $\frac{{\partial {\text{trait}}_{\text{i}} }}{{\partial {\bar{\text{I}}}\left( {{\text{R}}_{0} ,{\text{T}}} \right)}}$ is the slope of the reaction norm of production trait ${\text{i}}$, and $\frac{{\partial {\text{profit}}}}{{\partial {\text{trait}}_{\text{i}} }}$ is the economic value of production trait ${\text{i}}$. Under these assumptions, ${\text{L}}$ per host is a function of ${\text{R}}_{0}$:

6 $${\text{L}}\left( {{\text{R}}_{0} } \right) = {\bar{\text{I}}}\left( {{\text{R}}_{0} ,{\text{T}}} \right) \cdot {\text{L}}_{\text{par}} .$$

From Eqs. 1 and 6, it follows that:

7 $${\text{EV}} = \frac{{\partial {\text{L}}}}{{\partial {\text{R}}_{0} }} = \frac{{\partial \left( {{\bar{\text{I}}}\left( {{\text{R}}_{0} ,{\text{T}}} \right) \cdot {\text{L}}_{\text{par}} } \right)}}{{\partial {\text{R}}_{0} }} = {\text{L}}_{\text{par}} \cdot \frac{{\partial {\bar{\text{I}}}\left( {{\text{R}}_{0} ,{\text{T}}} \right)}}{{\partial {\text{R}}_{0} }},$$

8 $${\text{where}}\quad \frac{{\partial {\bar{\text{I}}}\left( {{\text{R}}_{0} ,{\text{T}}} \right)}}{{\partial {\text{R}}_{0} }} = {\text{I}}_{ \min } \cdot \frac{{\uptau \cdot {\text{R}}_{0}^{\uptau} \cdot \ln \left( {{\text{R}}_{0} } \right) - {\text{R}}_{0}^{\uptau} + 1}}{{{\text{R}}_{0} \cdot\uptau \cdot { \ln }\left( {{\text{R}}_{0} } \right)^{2} }}.$$

Equations 7 and 8 give the economic value of ${\text{R}}_{0}$ when expenditures are kept constant.

### Derivation of the economic value when losses are kept constant

When losses are kept constant, the economic value follows from Eq. 2. Thus, in this case we need to express ${\text{E}}$ as a function of ${\text{R}}_{0}$. Here, we assume that the only expenditures on disease control that change with ${\text{R}}_{0}$ consist of treatment costs. In the Discussion section, we show that partitioning ${\text{E}}$ into costs of treatment and prevention is irrelevant for the outcome. Thus, in the remainder of the paper, ${\text{E}}$ will refer to expenditures on treatment.

We assume that ${\text{E}}$ is linearly related to the number of treatments per production cycle with constant expenditures per treatment. The number of treatments per production cycle equals the length of a production cycle divided by the period between treatments. To keep ${\text{L}}$ constant, treatment must be applied when the number of parasites per host reaches a fixed maximum value. Thus, the period between treatments equals the time needed for the number of parasites per host to grow from its minimum just after treatment to the value at which treatment is applied. This means that the period between treatments is no longer fixed but has become a function of parasite growth rate, i.e. ${\text{R}}_{0}$. In other words, improvement of ${\text{R}}_{0}$ increases the period between treatments and decreases ${\text{E}}$. The corresponding algebra is provided below.

Let ${\text{E}}_{\text{treat}}$ be expenditures per treatment, and ${\text{I}}_{ \max }$ the number of parasites per host when treatment is applied. From Eq. 3 it follows that, for given values of ${\text{I}}_{ \max }$ and ${\text{I}}_{ \min }$, the period between treatments ($\uptau$) in parasite generations is:

9 $$\uptau\left( {{\text{R}}_{0} } \right) = \frac{{\ln \left( {{\text{I}}_{ \max } /{\text{I}}_{ \min } } \right)}}{{\ln \left( {{\text{R}}_{0} } \right)}}.$$

The number of treatments per production cycle of ${\text{T}}$ parasite generations equals ${\text{T}}/\uptau\left( {{\text{R}}_{0} } \right)$, from which it follows that:

10 $${\text{E}}\left( {{\text{R}}_{0} } \right) = \frac{\text{T}}{{\uptau\left( {{\text{R}}_{0} } \right)}} \cdot {\text{E}}_{\text{treat}} = {\text{T}} \cdot {\text{E}}_{\text{treat}} \cdot \frac{{\ln \left( {{\text{R}}_{0} } \right)}}{{\ln \left( {{\text{I}}_{ \max } /{\text{I}}_{ \min } } \right)}},$$

which gives ${\text{E}}$ as function of ${\text{R}}_{0}$. From Eqs. 2 and 10, it follows that:

11 $${\text{EV}} = \frac{{\partial {\text{E}}}}{{\partial {\text{R}}_{0} }} = \frac{{\partial \left( {{\text{T}} \cdot {\text{E}}_{{{\text{treat}}.}} \cdot \frac{{\ln \left( {{\text{R}}_{0} } \right)}}{{\ln \left( {{\text{I}}_{ \max } /{\text{I}}_{ \min } } \right)}}} \right)}}{{\partial {\text{R}}_{0} }} = \frac{{{\text{T}} \cdot {\text{E}}_{{{\text{treat}}.}} }}{{\ln \left( {{\text{I}}_{ \max } /{\text{I}}_{ \min } } \right)}} \cdot \frac{{\partial \ln \left( {{\text{R}}_{0} } \right)}}{{\partial {\text{R}}_{0} }} = \frac{{{\text{T}} \cdot {\text{E}}_{{{\text{treat}}.}} }}{{\ln \left( {{\text{I}}_{ \max } /{\text{I}}_{ \min } } \right)}} \cdot \frac{1}{{{\text{R}}_{0} }},$$

which gives the economic value of ${\text{R}}_{0}$ when losses are kept constant.

## Results

### Numerical example

**Fig. 2:**
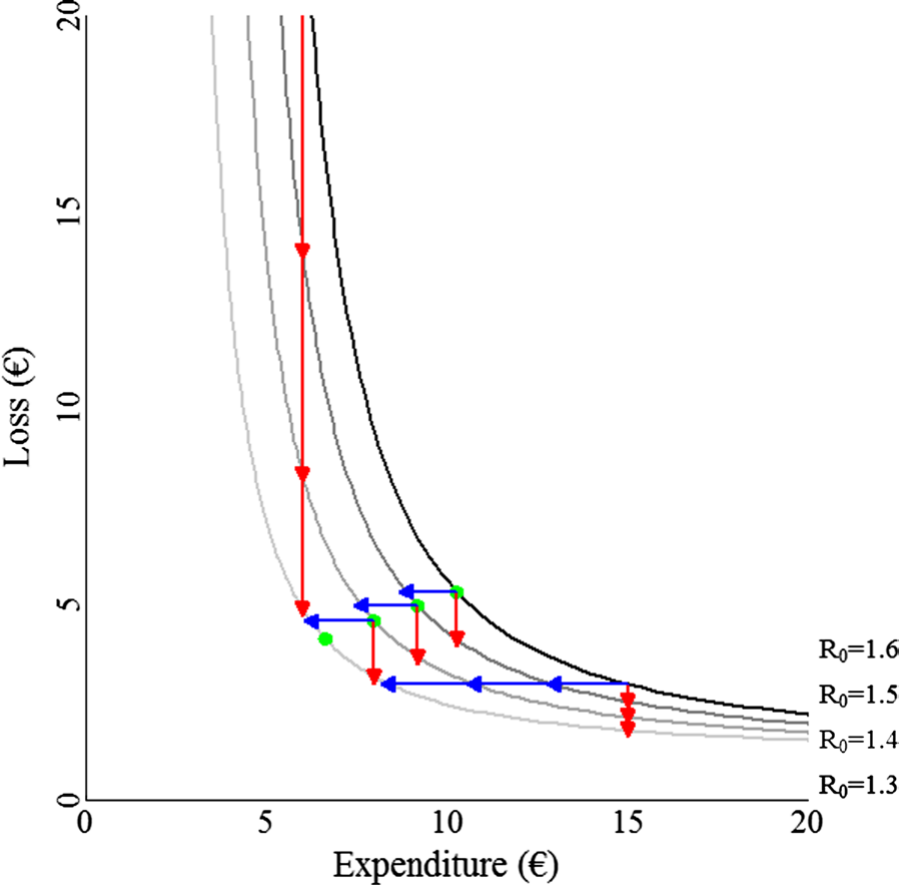
Loss-expenditure frontiers used in the numerical example. Red arrows (pointing downwards): reductions in losses with constant expenditures, blue arrows (pointing leftwards): reductions in expenditures with constant losses, green dots: optimum levels of expenditures

A numerical example with hypothetical loss-expenditure frontiers is provided to illustrate the economic effect of genetic improvement of ${\text{R}}_{0}$ (Fig. 2). The loss-expenditure curves are created by calculating ${\text{E}}$ from Eq. 10 and ${\text{L}}$ from Eq. 6 using the input parameters in Table 1. Red arrows (pointing downwards) illustrate reductions in ${\text{L}}$ with constant ${\text{E}}$, and blue arrows (pointing leftwards) illustrate reductions in ${\text{E}}$ with constant ${\text{L}}$.

**Table 1:** Input parameters for hypothetical loss-expenditure frontiers

Item	Symbol	Value	Unit
Minimum number of parasites per host	${\text{I}}_{ \min }$	1	Parasites/host
Length of a production cycle	T	12	Parasite generations
Time between two treatments	τ	0–120	Parasite generations
Losses per host per parasite present over a period of ${\text{T}}$ parasite generations	${\text{L}}_{\text{par}}$	1	€/parasite/host
Expenditures per treatment	${\text{E}}_{\text{treat}}$	5	€/treatment
${\text{R}}_{0}$	${\text{R}}_{0}$	1.3, 1.4, 1.5, and 1.6	

Figure 2 shows that when the level of ${\text{E}}$ is optimized before genetic improvement, the lengths of the red and blue arrows are similar. Those lengths are identical for a marginal change in ${\text{R}}_{0}$. For the loss-expenditure frontier where ${\text{R}}_{0} = 1.6$, the optimum frequency of treatment is once per 5.84 parasite generations ($\uptau = 5.84$). The economic value may be calculated either from the reduction in ${\text{L}}$ with constant ${\text{E}}$ (Eqs. 7 and 8):

12 $$\begin{aligned} {\text{EV}} & = \frac{{\partial {\text{L}}}}{{\partial {\text{R}}_{0} }} = {\text{L}}_{\text{par}} \cdot {\text{I}}_{ \min } \cdot \frac{{\uptau \cdot {\text{R}}_{0}^{\uptau} \cdot \ln \left( {{\text{R}}_{0} } \right) - {\text{R}}_{0}^{\uptau} + 1}}{{{\text{R}}_{0} \cdot\uptau \cdot \ln \left( {{\text{R}}_{0} } \right)^{2} }} \\ & = 1 \cdot 1 \cdot \frac{{5.84 \cdot 1.6^{5.84} \cdot \ln \left( {1.6} \right) - 1.6^{5.84} + 1}}{{1.6 \cdot 5.84 \cdot \ln \left( {1.6} \right)^{2} }} = \EUR 13.7 / {\text{unit}}\,{\text{R}}_{0}^{1} , \\ \end{aligned}$$

or from the reduction in ${\text{E}}$ with constant ${\text{L}}$ (Eq. 11)^1^:

13 $${\text{EV}} = \frac{{\partial {\text{E}}}}{{\partial {\text{R}}_{0} }} = \frac{{{\text{T}} \cdot {\text{E}}_{{{\text{treat}}.}} }}{{\ln \left( {{\text{I}}_{ \max } /{\text{I}}_{ \min } } \right)}} \cdot \frac{1}{{{\text{R}}_{0} }} = \frac{12 \cdot 10}{{\ln \left( {1 \cdot 1.6^{5.84} /1} \right)}} \cdot \frac{1}{1.6} = \EUR 13.7 / {\text{unit}}\,{\text{R}}_{0} .$$

As expected, both methods give identical results.

**Fig. 3:**
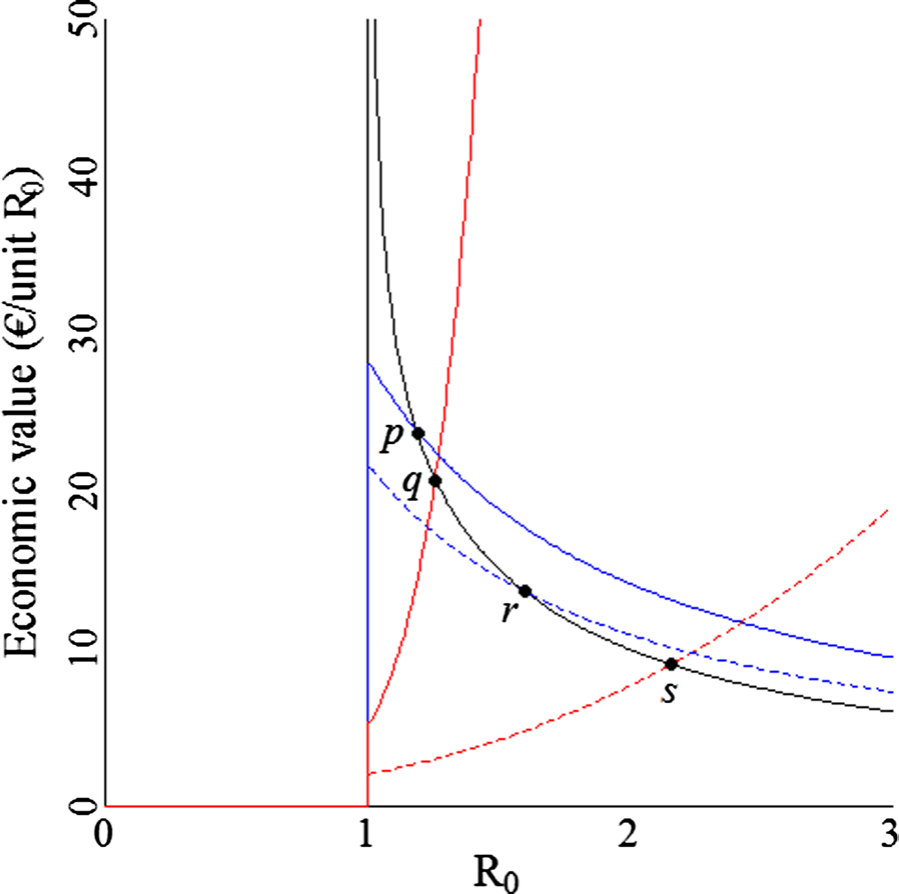
Economic values for a range of values for ${\text{R}}_{0}$ for different management strategies in the numerical example. Black line: optimized expenditures, red line: constant expenditures of €6, dashed red line: constant expenditures of €15, solid blue line: constant losses of €3.4, dashed blue line: constant losses of €5.3. Points *p*, *q*, *r*, and *s* are where a strategy with optimized expenditures results in the same economic value as strategies with constant expenditures or constant losses. Note that the minus sign in the economic value is ignored for presentation purposes

Relative differences in the lengths of the arrows in Fig. 2 are proportionate to relative differences in economic values, and depend on the value of ${\text{R}}_{0}$ and the management strategy. The management strategy may be to adopt the optimum level of ${\text{E}}$, or to reduce ${\text{L}}$ while ${\text{E}}$ is kept constant, or to reduce ${\text{E}}$ while ${\text{L}}$ is kept constant. When the level of ${\text{E}}$ is optimized before genetic improvement, the lengths of the red and blue arrows increase when ${\text{R}}_{0}$ decreases, thus the economic value increases when ${\text{R}}_{0}$ decreases. When ${\text{E}}$ is kept constant, the length of the red arrows decreases when ${\text{R}}_{0}$ decreases, thus the economic value decreases when ${\text{R}}_{0}$ decreases. When ${\text{L}}$ is kept constant, the length of the blue arrows increases when ${\text{R}}_{0}$ decreases, thus the economic value decreases when ${\text{R}}_{0}$ decreases. To illustrate these patterns, the economic value of ${\text{R}}_{0}$ is plotted as a function of the value of ${\text{R}}_{0}$ for the different management strategies (Fig. 3). Note that the economic value itself does not completely determine economic gain. Economic gain due to genetic improvement is the product of the economic value and genetic gain in ${\text{R}}_{0}$. The latter is expected to decrease with decreasing ${\text{R}}_{0}$.

The black line in Fig. 3 gives the economic value when ${\text{E}}$ is optimized, and shows that the economic value increases when the value of ${\text{R}}_{0}$ decreases. The economic value increases because both the mean number of parasites per host and the frequency of treatments decrease at an increasing rate when ${\text{R}}_{0}$ decreases (see Additional file 1: Fig S1).

The solid red line in Fig. 3 gives the economic value when ${\text{E}}$ is held constant at a level of €6, corresponding to the three red arrows on the left in Fig. 2. The dashed red line gives the economic value when ${\text{E}}$ is held constant at a level of €15, corresponding to the three red arrows on the right in Fig. 2. Both red lines show that, when ${\text{E}}$ is kept constant, the economic value decreases as ${\text{R}}_{0}$ decreases. Note that when ${\text{E}}$ is kept constant, the economic value is higher at lower levels of ${\text{E}}$ and decreases faster with decreasing values of ${\text{R}}_{0}$. The solid red line intersects the black line at point *q* where ${\text{R}}_{0} = 1.26$. At point *q* both economic values are equal, which implies that a level of ${\text{E}}$ at €6 is optimum for a ${\text{R}}_{0}$ value of 1.26. Because the optimum level of ${\text{E}}$ decreases when the value of ${\text{R}}_{0}$ decreases (Fig. 1a), the given level of ${\text{E}}$ (€6) is above the optimum for ${\text{R}}_{0} < 1.26$, whereas it is below the optimum for ${\text{R}}_{0} > 1.26$. Similarly, the dashed red line intersects the black line at point *s* where ${\text{R}}_{0} = 2.16$, thus a level of ${\text{E}}$ at €15 is optimum for a ${\text{R}}_{0}$ value of 2.16. Thus, the given level of ${\text{E}}$ (€15) is above the optimum for ${\text{R}}_{0} < 2.16$, whereas it is below the optimum for ${\text{R}}_{0} > 2.16$. The economic value when ${\text{E}}$ is kept constant below its optimum is higher than the economic value when ${\text{E}}$ is optimized, while the economic value is lower when ${\text{E}}$ is kept constant above its optimum.

The solid blue line in Fig. 3 gives the economic value when ${\text{L}}$ is held constant at a level of €3.4, corresponding to the three blue arrows at the bottom of Fig. 2. The dashed blue line gives the economic value when ${\text{L}}$ is held constant at a level of €5.3. Both blue lines show that, when ${\text{L}}$ is kept constant, the economic value increases when the value of ${\text{R}}_{0}$ decreases. The solid blue line intersects the black line at point *p* where ${\text{R}}_{0} = 1.19$. At point *p,* both economic values are equal, which implies that a level of ${\text{L}}$ at €3.4 is optimum for a ${\text{R}}_{0}$ value of 1.19. Because the optimum level of ${\text{L}}$ decreases when the value of ${\text{R}}_{0}$ decreases (Fig. 1a), the given level of ${\text{L}}$ (€3.4) is above the optimum for ${\text{R}}_{0} < 1.19$, whereas it is below the optimum for ${\text{R}}_{0} > 1.19$. Similarly, the dashed blue line intersects the black line at point *r* where ${\text{R}}_{0} = 1.60$, thus a level of ${\text{L}}$ at €5.3 is optimum for a ${\text{R}}_{0}$ value of 1.60. Thus, the given level of ${\text{E}}$ (€5.3) is above the optimum for ${\text{R}}_{0} < 1.60$, whereas it is below the optimum for ${\text{R}}_{0} > 1.60$. We consider the parts of the blue lines that are below the black line as irrelevant, because for these situations the economic value should follow from the reduction in ${\text{L}}$ with constant ${\text{E}}$ instead. The economic value when ${\text{L}}$ is kept constant below its optimum is higher than the economic value when ${\text{E}}$ is optimized.

To summarize the above, we can consider the following scenarios. When management is optimized, the economic value increases as ${\text{R}}_{0}$ decreases (black line). When ${\text{E}}$ is held constant above its optimum, the economic value is lower than when management is optimized. When ${\text{E}}$ is held constant below its optimum, the economic value is higher than when management is optimized. When ${\text{L}}$ is held constant below its optimum, the economic value is higher than when management is optimized.

A practical implication for continued genetic improvement may be that the management strategy shifts from a strategy where either ${\text{L}}$ or ${\text{E}}$ is held constant (red or blue lines) to the optimum strategy (black line). For example, management may operate under a constraint on ${\text{E}}$, due to which ${\text{E}}$ cannot exceed €6. At first when ${\text{R}}_{0}$ is larger than 1.26 (right of point *q*), management may reduce ${\text{L}}$ while ${\text{E}}$ is kept constant in response to genetic improvement. At some point (left of point *q*), ${\text{R}}_{0}$ may become smaller than 1.26, and management can adopt the optimum level of ${\text{E}}$, which will be below €6. Thus, in this example the economic value would first decrease following the solid red line in Fig. 3. As from when ${\text{R}}_{0} \le 1.26$ (point *q*), it would increase following the black line. Similarly, management may operate under a constraint on ${\text{L}}$, due to which ${\text{L}}$ cannot exceed €3.4. In this case, the economic value would first increase following the blue line. As from when ${\text{R}}_{0} \le 1.19$ (point *p*), it would increase following the black line.

### Example: sea lice in Norwegian salmon aquaculture

Sea lice are one of the major challenges in Norwegian salmon aquaculture. To protect wild salmon populations from infection by farmed salmon, sea lice numbers on farmed salmon are controlled by legislation. Treatment is obligatory when juvenile or adult female lice numbers exceed a threshold. Expenditures on treatment have been estimated at €0.087/treatment/kg production [6]. Ironically, treatment also induces production losses, which may even exceed production losses induced by lice themselves. Production losses induced by treatment include an elevated mortality and increased feed conversion ratio during and shortly after treatment. Total production losses induced by treatment have been estimated at €0.040/treatment/kg production [6]. Thus, each treatment involves a cost of €0.127/treatment/kg production. Moreover, current sea lice problems limit the expansion of the salmon farming industry in Norway. Solving these problems by genetic improvement of ${\text{R}}_{0}$ might increase production in the long run, which would further increase benefits of selection for R_0_. However, a detailed treatment of this issue is outside the scope of this paper.

To derive the economic value, we assume that the level of expenditures is above the economic optimum and that genetic improvement of ${\text{R}}_{0}$ reduces the frequency of treatments, while losses induced by lice are kept constant. For simplicity, a threshold for treatment on adult female lice only is considered here. Treatment efficacy is 95% [24], hence ${\text{I}}_{ \max } /{\text{I}}_{ \min }$ is 20. On average, salmon are treated 2.4 times per year [6]. A production cycle lasts about 500 days followed by a 60-day fallowing period. Thus, the average number of treatments per production cycle is 560/365 · 2.4 = 3.7. The generation interval of sea lice is about 70 days [24], hence ${\text{T}} = 500/70 = 7.1$ generations, $\uptau = {\text{T}}/3.7 = 1.9$ generations, and ${\text{R}}_{0} = \left( {\frac{{{\text{I}}_{ \max } }}{{{\text{I}}_{ \min } }}} \right)^{1/\tau } = 4.7$ (Eq. 9). ${\text{E}}_{\text{treat}}$ is €0.127/treatment/kg production. The economic value is (Eq. 11)^2^:

14 $${\text{EV}} = \frac{{{\text{T}} \cdot {\text{E}}_{\text{treat}} }}{{\ln \left( {{\text{I}}_{ \max } /{\text{I}}_{ \min } } \right)}} \cdot \frac{1}{{{\text{R}}_{0} }} = \frac{7.1 \cdot 0.127}{{\ln \left( {20} \right)}} \cdot \frac{1}{4.7} = 0.065\,\EUR / {\text{unit}}\,\,{\text{R}}_{0} \,\, / {\text{kg}}\,\,{\text{production}}.$$

We can use this value to quantify the economic importance of genetic improvement of ${\text{R}}_{0}$ for sea lice to the Norwegian salmon aquaculture industry. Consider, for example, the effect of one genetic standard deviation reduction in ${\text{R}}_{0}$. Selection against sea lice is generally based on dedicated and controlled challenge tests. Challenge tests are preferred over natural infections, because they allow standardization of testing protocols and avoid confounding affects when a subset of the challenged population have already acquired some degree of immunity due to prior exposure [25]. In a challenge test, fish are exposed to a high dose of copepodids (juvenile sea lice) and lice count per fish is recorded shortly after lice attachment. Part of the variation in lice count is determined by variation in skin surface area, which can be corrected for by using the allometric relation between body weight and skin surface area. The resulting trait is termed lice density [26]. Remaining variation in lice density is assumed to be explained by variation in susceptibility among individuals.

First, consider the case with genetic variation in susceptibility only. In this case, genetic variation in ${\text{R}}_{0}$ is proportional to genetic variation in susceptibility, which in turn is proportional to genetic variation in lice density. Thus, in this case breeding values for ${\text{R}}_{0}$ can be inferred directly from a challenge test. The genetic coefficient of variation of lice density is about 0.35 [26]. Thus, one genetic standard deviation improvement gives a 35% improvement in ${\text{R}}_{0}$, corresponding to 0.35 · 4.7 = 1.6 units ${\text{R}}_{0}$. Using the economic value of ${\text{R}}_{0}$ derived above, this improvement is expected to reduce expenditures on treatment by 1.6 · 0.065 = 0.11 €/kg production. For comparison, in 2017 the farm gate price of salmon was about €6.30/kg [27]. For the whole of Norway with a salmon production of 1.23 million tons [28], one standard deviation improvement in ${\text{R}}_{0}$ corresponds to a cost reduction of about 130 million €. In comparison, the expenditures on treatment and production losses induced by treatment combined are 380 million € [6].

The above analysis ignored genetic variation in infectivity among individuals. However, the result changes only when infectivity and susceptibility are genetically correlated; in the absence of such a correlation, selection for lice density does not yield a correlated response in infectivity. We might hypothesize that susceptibility and infectivity are positively correlated, because the same host-defence mechanisms may underlie these traits. For worm infections in sheep, for example, Kemper et al. [13] found that worm fecundity was reduced in sheep selected for low faecal egg count compared to a control line, where worm fecundity may be interpreted as a proxy for infectivity. A positive genetic correlation implies that selection for lower susceptibility via a challenge test induces a favourable correlated response in infectivity. Because susceptibility and infectivity have multiplicative effects on ${\text{R}}_{0}$ [14], the response in ${\text{R}}_{0}$ will then be more than proportionate to the response in susceptibility, and the benefits for industry will be larger than the figures presented above. To fully benefit from genetic variation in infectivity, breeding value estimation could be extended to include also infectivity, and estimated breeding values (EBV) of ${\text{R}}_{0}$ can be obtained by combining EBV for susceptibility and infectivity [14]. Estimation of breeding values for infectivity is challenging, but methods for that purpose have been developed [17, 18].

## Discussion

This study presents a method for the derivation of the economic value of ${\text{R}}_{0}$ for macroparasitic diseases. Economic values, even when imperfect [29], would improve the economic effectiveness of breeding programs. When ${\text{R}}_{0}$ ≤ 1, there is no risk for a major epidemic thus the economic value is zero. When ${\text{R}}_{0}$ > 1 and management is optimized, the economic value increases when ${\text{R}}_{0}$ decreases, because both the mean number of parasites per host and the frequency of treatments decrease at an increasing rate when ${\text{R}}_{0}$ decreases. Such an increase in economic value when ${\text{R}}_{0}$ decreases may be counter intuitive, because a decrease in ${\text{R}}_{0}$ will lower the sum of production losses and expenditures. However, the total costs of a disease are not a good proxy for the avoidable costs per unit genetic improvement of ${\text{R}}_{0}$. In line with these results, it is well-known in epidemiology that the effectiveness of vaccination programs increases when ${\text{R}}_{0}$ decreases [30]. When ${\text{R}}_{0}$ > 1 and management is not optimized, the economic value depends on whether genetic improvement is used for a reduction in expenditures or a reduction in production losses. When management is not optimized and ${\text{E}}$ or ${\text{L}}$ is held constant below its optimum, the economic value is higher than in optimized management. When management is not optimized and ${\text{E}}$ is held constant above its optimum, the economic value is lower than in optimized management. Because the relation between ${\text{R}}_{0}$ and farm profit is non-linear (Fig. 3), the economic value should be updated regularly for its actual value of ${\text{R}}_{0}$ [31]. For practical implementation in breeding programs, some directions are provided in the example on sea lice and in Anche et al. [14].

Although the aim of genetic improvement should not be to compensate for management inefficiencies [21], farmers may not always be able to operate under optimized management. For example, strict regulations on drug use apply in organic production [32]. Such regulations may constrain expenditures, forcing farmers to operate at a below optimum level of expenditures with relatively high losses. Genetic improvement would then reduce losses while expenditures are kept constant. In the example of sea lice in Norwegian salmon aquaculture, parasite numbers per host are constrained below the optimum, forcing farmers to operate at an above optimum level of expenditures with relatively low losses. In this case, genetic improvement reduces expenditures while losses are kept constant. The example also illustrates that genetic improvement is unlikely to reduce the infective pressure of sea lice on wild fish stocks in the short term, because mean lice numbers will not decrease when genetic improvement leads to a reduction in expenditures on treatment.

When losses are kept constant, the economic value of ${\text{R}}_{0}$ is derived from the reduction in expenditures. We assumed that expenditures for disease control consist of treatment costs only, but in reality they may also include preventive measures. In case of sea lice, preventive measures that reduce the overall infective pressure of lice may include the use of cleaner fish, lice skirts, and lasers. Here, we will show that the reduction in expenditures on treatment is equal to the reduction in the sum of expenditures on treatment and expenditures on preventive measures. Let ${\text{E}}\prime$ be the sum of expenditures on treatment (${\text{E}}_{\text{T}}^{\prime }$) and expenditures on preventive measures (${\text{E}}_{\text{P}}^{\prime }$). On the loss-expenditure frontier, the balance between ${\text{E}}_{\text{T}}^{\prime }$ and ${\text{E}}_{\text{P}}^{\prime }$ is optimum by definition, otherwise the level of ${\text{E}}\prime$ would be above the frontier. For any ${\text{L}}$, we could hypothesize an underlying ${\text{E}}_{\text{T}}^{\prime }$ − ${\text{E}}_{\text{P}}^{\prime }$ frontier. The level of ${\text{E}}\prime$ on the loss-expenditure frontier equals the sum of ${\text{E}}_{\text{T}}^{\prime }$ and ${\text{E}}_{\text{P}}^{\prime }$ at the point on the ${\text{E}}_{\text{T}}^{\prime }$ − ${\text{E}}_{\text{P}}^{\prime }$ frontier where $\partial {\text{E}}_{\text{T}}^{\prime } /\partial {\text{E}}_{\text{P}}^{\prime } = - 1$. Starting at this point, improvement of ${\text{R}}_{0}$ may reduce ${\text{E}}_{\text{T}}^{\prime }$ while ${\text{E}}_{\text{P}}^{\prime }$ and ${\text{L}}$ are kept constant or it may reduce ${\text{E}}_{\text{P}}^{\prime }$ while ${\text{E}}_{\text{T}}^{\prime }$ and ${\text{L}}$ are kept constant. For an infinitesimal improvement in ${\text{R}}_{0}$, the new ${\text{E}}_{\text{T}}^{\prime }$ − ${\text{E}}_{\text{P}}^{\prime }$ frontier between these points can be approximated linearly by $\partial {\text{E}}_{\text{T}}^{\prime } /\partial {\text{E}}_{\text{P}}^{\prime } = - 1$, hence $\partial {\text{E}}^{\prime }$ is constant between these points. Thus, based on the same reasoning as before, the economic value may be derived as the partial derivative of ${\text{E}}_{\text{T}}^{\prime }$ with respect to ${\text{R}}_{0}$, while ${\text{E}}_{\text{P}}^{\prime }$ and ${\text{L}}$ are held constant. This mathematical argument allows us to ignore the complex relation between expenditures on preventive measures and ${\text{R}}_{0}$. Another way to approach the issue is by considering a three-dimensional loss-expenditure frontier, where one axis represents production losses, one axis represents expenditures on treatment, and the third axis represents expenditures on preventive measures. We have ignored the axis on preventive measures based on the mathematical grounds given before, but we expect that the surface of the frontier is rather flat in this direction. We expect preventive measures to be a relative attractive control option compared to treatment, such that expenditures on preventive measures are not so responsive to genetic improvement of ${\text{R}}_{0}$. For example in case of sea lice, we would expect farmers to reduce rather the number of treatments than the use of cleaner fish. Still genetic improvement may lead to changes in expenditures on preventive measures that affect the value of ${\text{R}}_{0}$. Because, in this study, the method to derive the economic value ignores the effect of genetic improvement on preventive measures that affect ${\text{R}}_{0}$, the value of ${\text{R}}_{0}$ may not improve as much as expected. The estimated value of ${\text{R}}_{0}$ should therefore be evaluated regularly and the economic value should be updated accordingly.

In this study, the economic value of ${\text{R}}_{0}$ includes production losses due to disease. However, the breeding goal usually also includes yield as a trait. This introduces the risk of double-counting of production losses due to disease, which occurs when they are counted via the products of economic values and EBV of both ${\text{R}}_{0}$ and yield. To avoid double-counting, one might restrict the economic value of ${\text{R}}_{0}$ to expenditures on disease control, and include production losses due to disease in the economic value of yield. However, the economic value of yield would then include a non-linear component for production losses due to changing dynamics of disease transmission (Eq. 7) and a linear component for yield independent of production losses, which seems non-trivial. Furthermore, the effect of genetic improvement on management via reduction in losses or expenditures becomes unclear. These issues are resolved in the current study, where the economic value of ${\text{R}}_{0}$ includes production losses. As a consequence, we have to define yield to refer to individuals experiencing equal production losses due to disease (e.g. in the absence of disease). For the derivation of selection index weights and for prediction of the response to selection, it is essential that trait definitions agree between the breeding goal, the selection index, and the breeding value estimation. Thus, if the breeding goal includes ${\text{R}}_{0}$ (including production losses) and yield at equal production losses, then the selection-index weights and EBV should also refer to those same traits. Ideally, this is achieved by separate recording of phenotypes for production traits on animals that have equal production losses, while phenotypes to estimate breeding values for ${\text{R}}_{0}$ are recorded on a different group of animals. This situation is common in salmon breeding programs, where production traits are recorded in commercial conditions with small (and therefore similar) numbers of lice per fish, while susceptibility to sea lice is recorded in dedicated challenge tests. When phenotypes for production traits and phenotypes to estimate breeding values for ${\text{R}}_{0}$ are recorded instead on the same animals, phenotypes for production traits will include production losses. In sheep, for example, faecal egg count is recorded together with live weight on sheep maintained on infected pastures. Susceptible sheep have a relatively high faecal egg count and thus a lower live weight. To avoid double-counting of production losses in such a situation, the number of parasites per host may be included as a covariate in the linear model used for breeding value estimation for production traits. Using this approach, Bishop, et al. [33] estimated the slope of the regression of live weight on log-transformed faecal egg count to be − 1.28 kg/ln (faecal egg count).

In addition to ${\text{R}}_{0}$, other disease-related traits of potential interest for genetic improvement include tolerance and resilience. Tolerance is defined as an animal’s ability to cope with the effects of infection [34]. The economic importance of tolerance depends highly on disease status, which is determined by the value of ${\text{R}}_{0}$. Tolerance may be included in the breeding goal in addition to ${\text{R}}_{0}$, but the derivation of its economic value is beyond the scope of this study. Resilience is defined as an animal’s productivity in the face of infection [34], which is some sort of aggregate measure of ${\text{R}}_{0}$, tolerance, and production traits. As explained above, it does not seem wise to combine such different traits in a single measure.

A few other studies have attempted to derive economic values for macroparasitic disease traits, and some principles were discussed in general terms by Woolaston and Baker [35]. Woolaston and Baker [35] consider frequency of treatments and production losses as two separate and mutually exclusive breeding goal traits for macroparasitic diseases. Instead, we consider frequency of treatments and production losses as different management strategies that result from the underlying trait ${\text{R}}_{0}$. We have shown that both frequency of treatments (expenditures) and production losses may decrease in response to genetic improvement of ${\text{R}}_{0}$ when management is optimized. Including only one of these management variables as a trait in the breeding goal excludes the relevant scenario of optimized management. Bishop and Stear [36] demonstrated that the selection response for measures of disease prevalence cannot be predicted from quantitative genetic theory alone, because quantitative genetic theory disregards the underlying dynamics of disease transmission determined by ${\text{R}}_{0}$. Their findings are in agreement with the non-linear relations between ${\text{R}}_{0}$ and the number of parasites at any given time (Eq. 3), and between ${\text{R}}_{0}$ and the mean number of parasites between treatments (Eq. 4). Similarly, the response in frequency of treatments cannot be predicted from quantitative genetic theory alone, due to the non-linear relation between ${\text{R}}_{0}$ and frequency of treatments (Eqs. 9 and 10). The economic value may compensate for this bias in the predicted selection response resulting in the appropriate emphasis on the breeding goal trait, as in Amer et al. [37] for faecal egg count in sheep. In contrast, when ${\text{R}}_{0}$ is the breeding goal trait, the response to selection can be predicted from quantitative genetic theory only [14], such that the product of the response in units ${\text{R}}_{0}$ combined with the economic value of ${\text{R}}_{0}$ gives a direct prediction of the economic response to selection. Gharbi et al. [38] used an epidemiological model to describe the relation between genetic improvement and frequency of treatments for sea lice in salmon, but we have not been able to replicate their results. Lobo et al. [39] derived the economic value of the number of anthelmintic doses used per year for sheep. Bishop et al. [33] derived the economic value of faecal egg count for sheep from its negative effect on live weight. Neither Lobo et al. [39] nor Bishop et al. [33] explicitly considered whether genetic improvement would reduce production losses, expenditures, or both and neither of these studies considered that quantitative genetic theory fails to predict the response to selection for these traits. These issues would be resolved if ${\text{R}}_{0}$ was used as the breeding goal trait for which the economic value can be derived with the method outlined in this paper.

## Conclusions

This study presents a method for the derivation of the economic value of ${\text{R}}_{0}$ for macroparasitic diseases. When management is optimized, the economic value increases with decreasing values of ${\text{R}}_{0}$ (until the threshold of ${\text{R}}_{0} = 1$, where it drops to zero). When management is not optimized, the economic value depends on whether genetic improvement is used for reduced expenditures or production losses. For sea lice in salmon, the economic value is estimated to be 0.065 €/unit ${\text{R}}_{0}$/kg production.

## Additional file

**Additional file 1: Fig. S1.** Frequency of treatments (a) and the mean number of parasites per host (b) when the level of expenditures is optimized for the value of R_0_ in the numerical example. When the level of expenditures is optimized for the value of R_0_ in the numerical example, the frequency of treatments and the mean number of parasites per host both decrease at an increasing rate as R_0_ decreases. As a result, E and L both decrease at an increasing rate as R_0_ decreases, hence the economic value increases as R_0_ decreases.

## Footnotes

1 Throughout the text, the minus sign in the economic value is ignored for presentation purposes.

2 Throughout the text, the minus sign in the economic value is ignored for presentation purposes.
