# Supplementary figures and images for: Derivation of the economic value of R0 for macroparasitic diseases and application to sea lice in salmon
Source: Genet Sel Evol. 2018 Oct 3;50:47. doi: 10.1186/s12711-018-0418-6 (PMC6171287; doi:10.1186/s12711-018-0418-6)

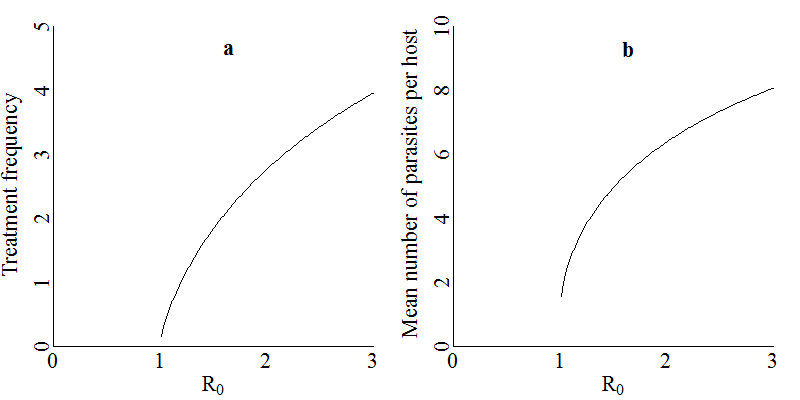

Supplement: Supplementary file 1 — Additional file 1: Fig. S1. Frequency of treatments (a) and the mean number of parasites per host (b) when the level of expenditures is optimized for the value of R0 in the numerical example. When the level of expenditures is optimized for the value of R0 in the numerical example, the frequency of treatments and the mean number of parasites per host both decrease at an increasing rate as R0 decreases. As a result, E and L both decrease at an increasing rate as R0 decreases, hence the economic value increases as R0 decreases. [file 12711_2018_418_MOESM1_ESM.docx]
